# The HTPmod Shiny application enables modeling and visualization of large-scale biological data

**DOI:** 10.1038/s42003-018-0091-x

**Published:** 2018-07-05

**Authors:** Dijun Chen, Liang-Yu Fu, Dahui Hu, Christian Klukas, Ming Chen, Kerstin Kaufmann

**Affiliations:** 1 Department for Plant Cell and Molecular Biology, Institute for Biology, Humboldt-Universität zu Berlin, Berlin, 10115 Germany; 2 Leibniz Institute of Plant Genetics and Crop Plant Research (IPK), Corrensstrasse 3, Gatersleben, 06466 Germany; 3 Department of Bioinformatics, College of Life Sciences, Zhejiang University, Hangzhou, 310058 China; 4 Present Address: Digitalization in Research & Development (ROM), BASF SE, Ludwigshafen am Rhein, 67056 Germany

## Abstract

The wave of high-throughput technologies in genomics and phenomics are enabling data to be generated on an unprecedented scale and at a reasonable cost. Exploring the large-scale data sets generated by these technologies to derive biological insights requires efficient bioinformatic tools. Here we introduce an interactive, open-source web application (HTPmod) for high-throughput biological data modeling and visualization. HTPmod is implemented with the Shiny framework by integrating the computational power and professional visualization of R and including various machine-learning approaches. We demonstrate that HTPmod can be used for modeling and visualizing large-scale, high-dimensional data sets (such as multiple omics data) under a broad context. By reinvestigating example data sets from recent studies, we find not only that HTPmod can reproduce results from the original studies in a straightforward fashion and within a reasonable time, but also that novel insights may be gained from fast reinvestigation of existing data by HTPmod.

## Abstract (web summary)

Dijun Chen et al. present HTPmod, a Shiny web application for modeling and visualization of large-scale genomic and phenomic datasets. The authors show that HTPmod can quickly reproduce analyses of high-throughput biological datasets and produce publication-quality figures.

## Introduction

Over the last decade, technological advances in genomics (e.g., high-throughput sequencing, HTS) and phenomics (high-throughput plant phenotyping, HTP) have resulted in a tremendous increase of molecular and phenotypic data from large number of samples with a high-dimensional list of measurements. As a result, we can acquire an extensive range of phenotypes at organism-wide scale^1,2^, quantify the expression of tens of thousands of genes^3–5^, and measure the entire epigenome^6,7^ or regulatome^8–10^ simultaneously for hundreds to thousands of samples at a reasonable cost. The immense volume, variety, velocity, and veracity of high-throughput biological data generated by these technologies make it a big data problem^11–13^. In this regard, data handling and processing remain a major technical bottleneck when translating big biological data into knowledge.

Extracting hidden patterns and making accurate predictions from these massive data sets largely rely on machine-learning approaches^14,15^. From a computational point of view, machine learning methods are attractive in terms of their ability to derive predictive models without a need for strong assumptions about underlying mechanisms; hence they are especially useful to deal with certain biological questions of which our a priori knowledge is frequently unknown or insufficiently defined^14^. As a proof of concept, gene expression levels can be accurately predicted from a broad set of epigenetic features^16–20^ or binding profiles of diverse transcription factors (TFs)^21–24^ using various machine-learning-based approaches, although our knowledge about how the selected features determine the expression output is largely unknown. Modeling is, therefore, a key ingredient to derive novel biological insights by integrating large-scale data sets. Generally, a canonical machine learning workflow consists of the model fitting and evaluation. Although conceptually simple, applying adequate machine-learning algorithms to the large corpus of data remains an important challenge since it requires substantial computational expertise and effort. To our knowledge, an integrative web-based application for interactive exploration and interpretation of large-scale, high-dimensional data sets is not available to date. Here we present an interactive web application, HTPmod (http://www.epiplant.hu-berlin.de/shiny/app/HTPmod/), for high-throughput biological data modeling and visualization. By reinvestigating example data sets from recent studies, we demonstrate that HTPmod can be used for modeling and visualizing multiple types of omics data (such as phenomics, transcriptomics, metabolomics, and epigenomics data) under a broad context in a straightforward and an efficient fashion.

## Results

### Overview of the HTPmod application

**Fig. 1:**
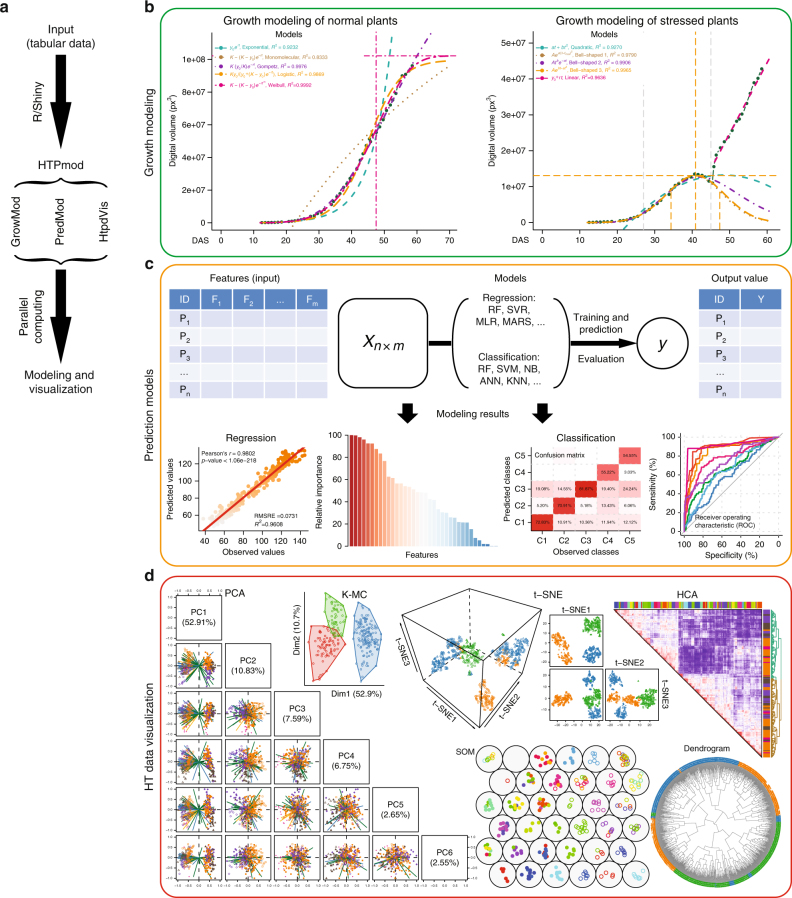
The HTPmod Shiny application for high-throughput data modeling and visualization. **a** The overall design and workflow of HTPmod. **b** The *growMod* module for plant growth modeling. Example results shown here are based on data from ref. ^1^. **c** The *predMod* application for predicting traits of interest from high-dimensional data using various prediction models. The upper panel shows the general workflow of *predMod*. The lower panel shows example output of regression (left) or classification (right) from *predMod*. **d** High-throughput data visualization with the *htpdVis* application. Example graphs are generated by *htpdVis* using data from refs. ^1,25^

By integrating existing machine-learning approaches applied in high-throughput experiments^1,25,26^, HTPmod was implemented with the Shiny framework (http://shiny.rstudio.com/), which combines the computational power of R with friendly and interactive web interfaces. HTPmod provides three function modules for modeling (*growMod* and *predMod*) and visualizing (*htpdVis*) data especially from high-throughput experiments, such as HTP and HTS (Fig. 1 and Supplementary Fig. 1). Besides, HTPmod accepts the simplest table files as the only input (Fig. 1a and Supplementary Fig. 2) and supports the generation of various types of publication-quality graphics (Fig. 1b–d) and tables with possible customizations. Whenever possible, HTPmod adopts parallel computing to speed up analysis.

### The *growMod* module for plant growth modeling

The first module in HTPmod, *growMod*, was developed for plant growth modeling based on time-series data, e.g., from plant HTP experiments^1,27^. HTP is an ideal tool to study plant growth in a noninvasive way. We previously showed that the growth of barley (*Hordeum vulgare*) plants under normal and drought stress growth conditions follows a logistic curve and a bell-shaped curve, respectively^1^. In this study, we provided a graphical user interface (GUI) to perform growth modeling in an easy and efficient way (Fig. 1b). Generally, input data for *growMod* can be extracted from images by existing HTP image analysis software, such as IAP^28^ or PlantCV^27,29^. Image-derived features, such as plant height, project area and digital volume are some examples of traits that can be used to model plant growth. The *growMod* tool supports growth modeling for normal and stressed plants, which can be done either at single plant level or at group level (i.e., replicates in a group or a genotype). Moreover, we included several mechanistic growth models (including linear, bell-shaped, quadratic, exponential, monomolecular, logistic, Weibull and Gompertz curves; Supplementary Table 1) so that the performance of each model can be compared and evaluated (see Methods). Users can choose proper growth models to predict plant growth in their studies. Finally, biologically interpretable parameters can be derived from these models and can be further used for association mapping in a large population, allowing a deeper understanding of the performance and genetic basis of plant growth^1^.

### The *predMod* module for prediction

The second module *predMod* was implemented with several supervised machine-learning models to relate input features (e.g., image data from HTP, and TF binding and histone modification data from HTS) to output quantities of interest (e.g., plant biomass, yield, stress status, or gene expression levels). The *predMod* tool is typically useful in situations where large amounts of data are available, with the aim to understand how a combination of factors (inputs) influence the output trait. In particular, the prediction models can be used for either regression (where output consists of numeric values) or classification (where output is a categorical class label). For instance, such prediction models have been widely used to predict the contribution of chromatin features to the change of gene expression^18,21,30^, to predict plant biomass from image-derived features^25,27,31^, to classify plants in different stress status^1^ or disease status^32^ based on image data, or to discriminate organ-specific target genes based on SELEX-seq data^26^. We integrated more than 30 widely used machine-learning approaches (Supplementary Table 2) into the *predMod* module, for regression or classification analyses (Fig. 1c). The prediction performance can be evaluated when multiple prediction models are selected^18,25,30^ (see Methods). Furthermore, feature importance and their prediction power can be extracted from the models^18,21,25,30^, which may aid for feature selection (e.g., to find potentially interesting features).

### The *htpdVis* module for visualization

However, when there is no prior knowledge of the data investigated, unsupervised machine-learning approaches can be used to discover patterns from large data sets. To this end, we developed a third module, *htpdVis*, to explore and visualize large-scale, high-dimensional data using various unsupervised machine-learning approaches, such as principal component analysis (PCA), t-distributed stochastic neighbor embedding (t-SNE)^33^, self-organizing map, multidimensional scaling, K-means clustering or hierarchical cluster analysis with heatmaps (Fig. 1d). This module is particularly useful for exploration of hidden patterns and exploratory data mining from omics data sets such as phenome^1^, transcriptome^34–36^, or epigenome data^37^. For example, in PCA, the results of top principal components (PCs) are usually shown in a scatterplot where both the component scores (the transformed variable values of data points) and the factor loadings (the correlation coefficients between the observations [rows] and factors or features [columns]) are plotted in the same graphs (Fig. 1d). In addition, we also implemented the PCA with self-organizing map clustering approach, which is a useful way to visualize and explore multidimensional data sets, such as gene expression data across tissues in multiple species^38–40^. Notably, in the *htpdVis* module, different parameter settings can be used to generate diverse types of graphs with color and shape schema highlighting important data features (Fig. 1d).

### Applications of HTPmod

To demonstrate the universal applications of HTPmod in data exploration and visualization, we provided various example data sets from recent studies (Supplementary Table 3) spanning phenomics^1,25,27^, metabolomics^41^, epigenomics^37^, regulatomics^21,26^ and transcriptomics^42^. We explored these data using the various functionalities implemented in our HTPmod system (see also online application for demonstrations). We showed that not only can HTPmod reproduce the corresponding findings of the original studies but also can gain novel insights from existing published data in a straightforward fashion and within a reasonable time (Supplementary Figs. 3-13).

**Fig. 2:**
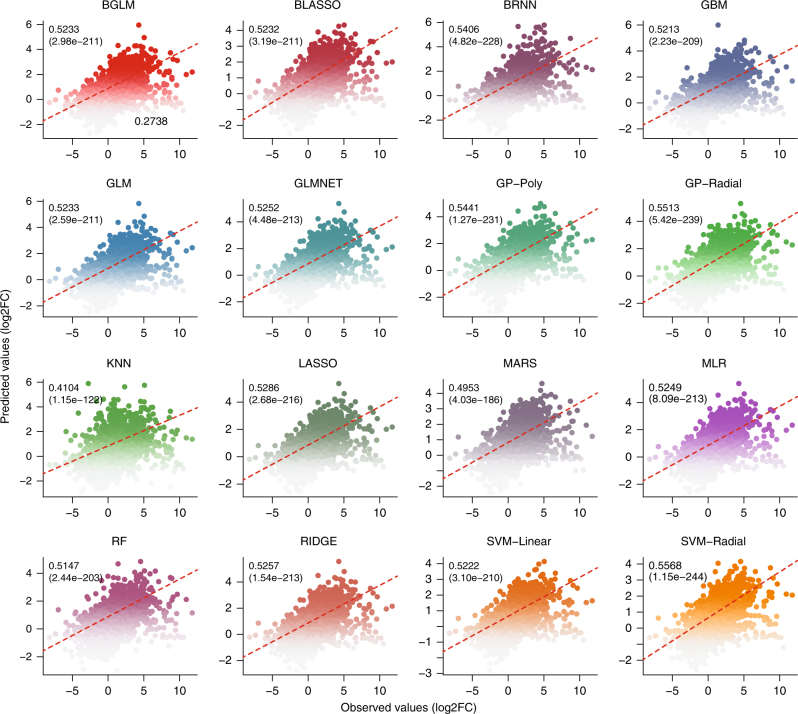
Prediction of gene expression changes using transcription factor binding data in Arabidopsis. Data obtained from ref. ^21^ and the full names of models referred to Supplementary Table 2. All prediction models with default parameter settings in *predMod* were used in the analysis. Pearson’s correlations and corresponding *p*-values (in parentheses) are shown

**Fig. 3:**
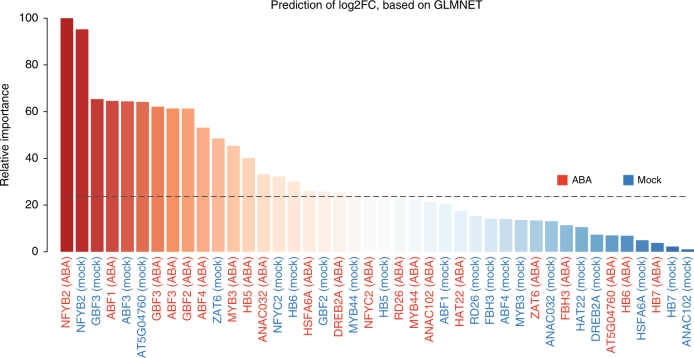
Relative importance of features in prediction of gene expression changes. GLMNET (lasso and elastic-net regularized generalized linear model) regression model (in *predMod*) was used to predict gene expression changes, using binding strength in both ABA- and mock-treated conditions. Barplot shows the relative importance of the binding features in the prediction. The result is consistent with that from the original study^21^

Here, we briefly described two case studies to show the power of HTPmod in data modeling and visualization. The first case study is to predict gene expression patterns using TF binding data in *Arabidopsis thaliana*, as shown in a recent study^21^. Briefly, we collected gene expression data from the supplemental data of ref. ^21^. and TF binding profiles from the Gene Expression Omnibus (GEO) database with an accession number GSE80568. The input data (consisting a matrix of TF binding score and expression changes for the differentially expressed genes) for HTPmod were prepared in a similar way as Song et al.^21^. We ran the *predMod* module with 16 regression models to relate TF binding strength to gene expression changes (log-transformed fold change [FC]) under ABA (phytohormone abscisic acid) treatment compared to mock. Strikingly, all the tested models show relatively comparable performance (Fig. 2 and Supplementary Fig. 7), implying that these models capture the intrinsic determinant of TF binding to the gene expression outcome. In addition, the relative feature importance determined by a glmnet regression model (Fig. 3) is consistent to the results presented in the original study^21^.

**Fig. 4:**
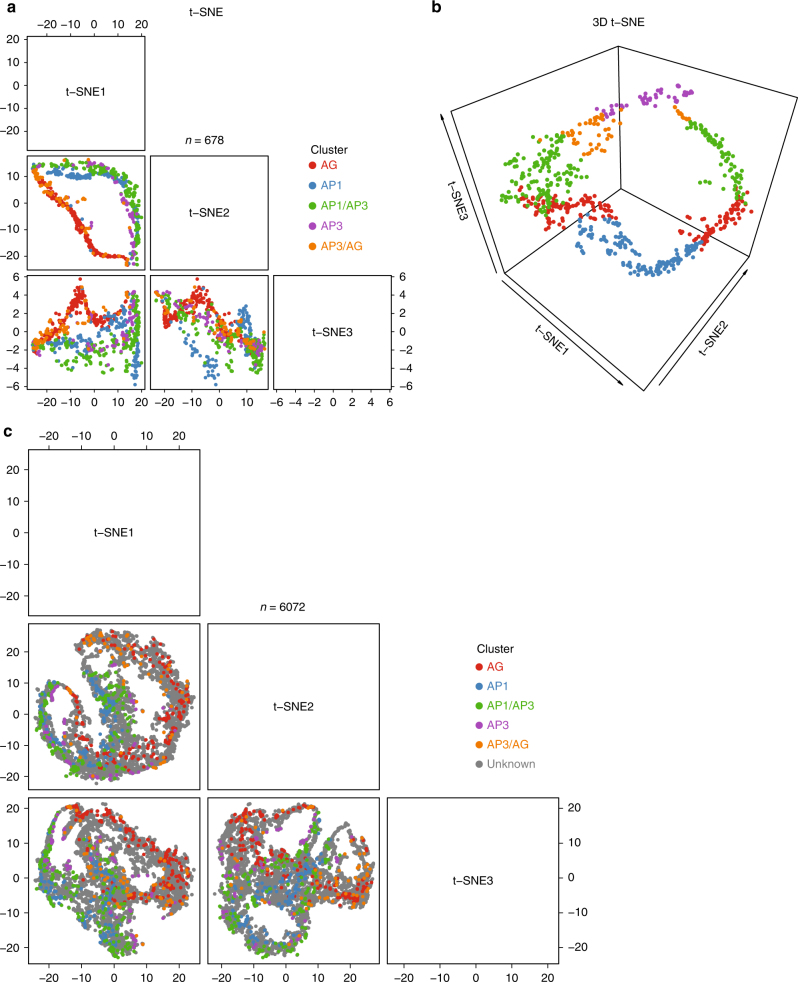
Visualization of floral organ-specific transcriptome data in *Arabidopsis*^42^ via t-SNE plots^33^ using *htpdVis*. The pattern of organ-specific expression for genes with known organ signature is shown in the three-dimensional t-SNE plots in 2D (**a**) or 3D (**b**) views. **c** t-SNE plot in 2D view showing organ-specific expression pattern by adding more genes with unknown organ signature. Default parameter settings were used in all of these analyses

The second case study is to visualize floral organ-specific gene expression patterns^42^ by the *htpdVis* module. Domain-specific translatome data were obtained from the supplemental file of ref. ^42^. Based on analysis of variance (ANOVA), we identified 6072 genes that show significant spatiotemporal domain effects (*p*-value <0.05 based on ANOVA) with at least two-fold change (FC > 2) between different domains. We then filtered 678 domain-specific genes (see online document for more details) that were highly expressed in AP1-specific (specifying the sepal organ), AG-specific (carpel), AP1/AP3-common (petal), or AP3/AG-common (stamen) domains. We projected the data onto three dimensions via t-SNE plots based on *htpdVis* (Fig. 4a, b), which confirms that these organ-specific genes show well defined, distinct expression pattern. When adding more genes with unknown organ signature into visualization, we observed spatiotemporal gene expression trajectories during floral organ development (Fig. 4c). These observations provide an important starting point to investigate the mechanisms regulating organ differentiation in plants. In summary, the above results strongly support that HTPmod can make fast reproducible analysis without any programming demand.

## Discussion

In this work, we developed and characterized a web application for modeling and visualizing large-scale biological data sets. As implemented with the Shiny framework, the HTPmod application inherits the computational power as well as professional visualization of R. To avoid excessively long run-times, HTPmod also allows parallel computing to speed-up analysis whenever possible, facilitated by the BiocParallel package (http://bioconductor.org/packages/release/bioc/html/BiocParallel.html). The BiocParallel allows parallelization either on local web machine or on a cluster of computers using specific job schedulers. In short, HPTmod offers three modules (*growMod*, *predMod*, and *htpdVis*) for exploratory or interactive data mining with various omics data sets. An obviously distinctive feature of HTPmod is that it integrates widely used mathematical models (Supplementary Table 1) and machine-learning approaches (Supplementary Table 2) and runs them in a uniform way on a single data set, therefore allowing direct comparison and evaluation of the performance of different methods. However, different models may show distinct performance for a specific data set. In this respect, we may choose a model of interest or a model with the best performance in the analysis. Furthermore, model-derived knowledge, such as parameters to describe plant growth and performance^1^, and feature importance scores^18,20,25^, may allow important biological interpretation and be promising for providing novel insights.

In order to demonstrate that HTPmod is powerful for modeling and visualization of large-scale biological data in different contexts, we provided several case studies ranging from genomics to phenomics^1,21,25–27,37,41,42^ (Supplementary Table 3) and have shown that HTPmod is an easy-to-use tool that generates reproducible results in a very efficient way. Compared to existing analysis protocols^38,43,44^, HTPmod offers several advantages. First of all, HTPmod provides user friendly web interfaces to run a diverse set of models for data modeling and visualization based on a single input file, thus without the need of programming experience. Second, HTPmod can generate a variety of plots for publication purposes based on a single data set. Finally, HTPmod is open source and highly extendable. New prediction models can be easily integrated into HTPmod (see the online document). We will continue to integrate more prediction models or visualization/analysis components in the future. For example, deep learning is an emerging approach in the field of machine learning that can be used for image-based analytical tasks in plant phenotyping^45–47^. We believe that the data organization and visualization features offered by HTPmod are valuable for data scientists trying to apply deep learning to their HTP images.

As more and more big genomic and phenomic data sets are being or are going to be generated by large-scale, high-throughput experiments, the methodological framework for data modeling and visualization proposed in this work will have broadly potential applications. We anticipate that the plentiful output generated by HTPmod on a single data set will be useful to advance our views of a specific biological question under investigation. In summary, HTPmod is an open-source, interactive, and powerful web platform for large-scale biological data modeling and visualization.

## Methods

### Growth modeling (*growMod*)

With HTP data, image-derived features like plant height, projected area^27^ and digital volume^1^ can be considered as growth-related traits for growth modeling. In the *growMod* module, plant growth in control conditions can be modeled with six different mechanistic models: linear, exponential, monomolecular, logistic, Gompertz, and Weibull models (Supplementary Table 1). In order to fit these models using the linear regression function “lm” in R, the non-linear relationship of the models were first transformed into linearized forms (Supplementary Table 1). The growth traits are then fitted with the linearized models. Finally, the performance of models is assessed and compared based on their *R*^2^ and *p*-values. Some useful parameters can be derived from these models. For example, for the logistic model, the following three parameters are important to describe plant growth performance:^1^ (1) the intrinsic growth rate (*R*) that measures the speed of growth; (2) the inflection point (IP) that represents the time point when plant reaches the maximal speed of growth; and (3) the maximum final vegetative biomass (*K*_max_), which was estimated for each plant on the basis that the model could fit the data with the largest *R*^2^.

We also implemented several models to predict plant growth in in drought stress conditions^1^ (Supplementary Table 1). The modeling steps are divided into two parts: (1) growth before and during the stress phase and (2) re-growth during recovery phase. In the first phase, three different bell-shaped curves and a quadratic curve are fitted to the data, while in the recovery phase a simple linear model is used to characterize re-growth with the speed of re-growth (*R*_rec_).

### Prediction models (*predMod*) for regression or classification analysis

We included 32 widely used machine-learning approaches (Supplementary Table 2) into the *predMod* module, for regression or classification analysis purposes. Based on the powerful functionality of the *caret* R package and the uniform criteria for model performance evaluation (see below), *predMod* enables to run these models in a similar manner with comparable output.

### Model performance

To evaluate the performance of the predictive models, we adopted a *k*-fold cross-validation strategy to check the prediction power of each model. Specifically, each data set will be randomly divided into a training set ((*k* − 1)/*k* of individuals) and a testing set (1/*k* of individuals). A specific model is first trained on the training data and then applied to make prediction for the testing data. The final performance of models is evaluated and compared based on the average prediction accuracies obtained from *N* resampling of the data set (*N*-times randomization), where both *k* and *N* are defined by users.

For regression models, their predictive performance can be measured by the Pearson correlation coefficient (PCC; *r*) between the predicted values and the observed values; and the coefficient of determination (*R*^2^) which equals to the fraction of variance explained by the model, defined as

$$R^2 = 1 - \frac{{{\mathrm {SS}}_{\mathrm {res}}}}{{{\mathrm {SS}}_{\mathrm {tot}}}} = 1 - \frac{{\mathop {\sum }\nolimits_{i = 1}^n \left( {y_i - \hat y_i} \right)^2}}{{\mathop {\sum }\nolimits_{i = 1}^n \left( {y_i - \bar y} \right)^2}}$$

where SS_res_ and SS_tot_ are the sum of squares for residuals and the total sum of squares, respectively, $\hat y_i$ the predicted and *y*_*i*_ the observed value of the *i*th plant, $\bar y$ is the mean value of the observed values; and the root mean squared relative error of cross-validation, defined as

$${\mathrm {RMSRE}} = \sqrt {\frac{{\mathop {\sum }\nolimits_{i = 1}^s \left( {\frac{{y_i - \hat y_i}}{{y_i}}} \right)^2}}{s}}$$

where *s* denotes the sample size of the testing data set.

We repeated the cross-validation procedure ten times. The mean and standard deviation of the resulting *R*^2^ and RMSRE values were calculated across runs.

The predictive bias *μ* between the predicted and observed values, defined as

$$\mu = \frac{1}{n} \cdot \mathop {\sum }\limits_{i = 1}^n \frac{{\hat y_i - y_i}}{{y_i}}$$

where *n* denotes the sample size of the data set. This bias indicates overestimation (*μ* > 0) or underestimation (*μ* > 0) of the target feature.

For classification models, their predictive performance can be measured by: (1) a confusion matrix, which is the contingency table of actual versus predicted class labels for each class, and is particularly helpful in the case of multiclass classification; (2) scalar characteristics as the accuracy, and average area under the ROC curve (see below); (3) a receiver operating characteristic (ROC) curve by plotting the true positive rate (TPR) against the false-positive rate (FPR) at various threshold settings, which is particularly helpful in two class problems; (4) a precision-recall curve (PRC)^48^ showing the tradeoff between precision and recall at different thresholds, which is particularly useful when the classes are very imbalanced.

### Influence of features on prediction performance

We also developed several criteria to evaluate the relative importance of features for the prediction. For the models (including random forest, stochastic gradient boosting, classification and regression trees and multivariate adaptive regression spline) with built-in strategies to estimate the contribution of each variable to the prediction, the estimated measures of relative importance are scaled to the range between 0 (least important) and 100 (most important). Otherwise, the importance of each predictor is calculated individually using a filter approach as implemented in the *caret* R package.

Furthermore, the following criteria are also used to evaluate the importance of individual features and their redundancy in prediction. For regression, the ability of individual features to predict the response variable is calculated as the correlation coefficients (*R*^2^) between the predicted values and the actual values, which is termed as predictive power of the corresponding features. For classification problems, a greedy feature selection algorithm^49^ is conducted. Specifically, starting with the original set of *n* features, each feature is independently removed to produce *n* subsets of data with *n* − 1 features. Then the classification performance is computed with *k*-fold cross-validation and *N*-times randomizations, in the same way as described above, for each of these *n* subsets. The feature with least decreased the classification accuracy will be removed at this step. The above process is iterated until no feature can be removed. The classification performance driven by a specific combination of features can be visualized in a boxplot, with *x*-axis as the number of features and y-axis as cross-validation of classification accuracy.

### Code availability

The HTPmod web-based application is freely available at http://www.epiplant.hu-berlin.de/shiny/app/HTPmod/. Users are encouraged deploy the HTPmod application at their own web server. The corresponding source code is available at https://github.com/htpmod/HTPmod-shinyApp and online document is available at https://github.com/htpmod/HTPmod-shinyApp/wiki.

### Data availability

The processed example data sets used for demonstration purposes are provided alongside the HTPmod source code (https://github.com/htpmod/HTPmod-shinyApp).

## Electronic supplementary material

Supplementary Information
