# Supplementary material for: The HTPmod Shiny application enables modeling and visualization of large-scale biological data
Source: Commun Biol. 2018 Jul 5;1:89. doi: 10.1038/s42003-018-0091-x (PMC6123733; doi:10.1038/s42003-018-0091-x)
Supplement: Supplementary file 1 — Supplementary Information [file 42003_2018_91_MOESM1_ESM.pdf]

Supplementary Tables

Supplementary Table 1. Mechanistic models used for growth modeling in *growMod*, modified from ref[1].

	Model	Differential equation†	Analytical solution†	Linearized form†
Control plants	**Exponential**	$\frac{\mathrm{d}y}{\mathrm{d}t} = ry(t)$	$y = y_0 e^{rt}$	$\ln(y) = \ln(y_0) + rt$
	**Monomolecular**	$\frac{\mathrm{d}y}{\mathrm{d}t} = r(K - y(t))$	$y = K - (K - y_0)\, e^{-rt}$	$\ln \frac{1}{K - y} = \ln \frac{1}{K - y_0} + rt$
	**Gompertz**	$\frac{\mathrm{d}y}{\mathrm{d}t} = ry(t) \ln \frac{K}{y(t)}$	$y = K \left(\frac{y_0}{K}\right)^{e^{-rt}}$	$-\ln\left(-\ln \frac{y}{K}\right) = -\ln\left(-\ln \frac{y_0}{K}\right) + rt$
	**Logistic**§	$\frac{\mathrm{d}y}{\mathrm{d}t} = ry(t)\left(1 - \frac{y(t)}{K}\right)$	$y = \frac{K y_0}{y_0 + (K - y_0)\, e^{-rt}}$	$\ln \frac{y}{K - y} = \ln \frac{y_0}{K - y_0} + rt$
	**Weibull**¶	$\frac{\mathrm{d}y}{\mathrm{d}t} = rmt^{m-1}(K - y(t))$	$y = K - (K - y_0)\, e^{-rt^m}$	$\ln\left(\ln \frac{K - y_0}{K - y}\right) = \ln(r) + m\ln(t)$
Stressed plants	**Quadratic**	$\frac{\mathrm{d}y}{\mathrm{d}t} = b - 2at$	$y = c + bt - at^2$	$y = c + bt - at^2$
	**Bell-shaped 1**	$\frac{\mathrm{d}y}{\mathrm{d}t} = 2Aa(t - t_{max})\, e^{a(t - t_{max})^2}$	$y = Ae^{a(t - t_{max})^2}$	$\ln(y) = \ln(A) + a(t - t_{max})^2$
	**Bell-shaped 2**	$\frac{\mathrm{d}y}{\mathrm{d}t} = A(b/t - a)\, t^b e^{-at}$	$y = At^b e^{-at}$	$\ln(y) = \ln(A) + b\ln(t) - at$
	**Bell-shaped 3**	$\frac{\mathrm{d}y}{\mathrm{d}t} = A(b - 2at)e^{bt - at^2}$	$y = Ae^{bt - at^2}$	$\ln(y) = \ln(A) + bt - at^2$
	**Linear**‡	$\frac{\mathrm{d}y}{\mathrm{d}t} = r$	$y = y_0 + rt$	$y = y_0 + rt$

† y is biomass; t denotes time; r is intrinsic growth rate for control plants or re-growth rate for stressed plants; K is upper asymptote of biomass for control plants in monomolecular, Gompertz, logistic and Weibull models; m determines the slope of growth in Weibull model; $t_{max} = \frac{b}{2a}$ is the time point (the center of the peak in bell-shaped curves) at which plant under stress shows the asymptotic maximum biomass (determined by A). Other parameters are constants.

§ Only the three-parameter version of logistic model was considered. In this model, the lower asymptote is fixed at 0 and the inflection point falls strictly at $y = K/2$.

¶ Weibull model with three parameters was considered, where $y_0 = 0$. The model can thus be simplified as $y = K\left(1 - e^{-rt^m}\right)$. It is reasonable in most cases. For example, at planting, the plant biomass is very close to zero (Archontoulis and Miguez, 2013).

‡ Linear growth for stressed plants is only modeled in the recovery phase.

Supplementary Table 2. Various machine-learning approaches integrated into HTPmod.

ID	Short name	Full name	R package[*]
Regression Models			
**1**	BGLM	Bayesian generalized linear model	*arm*
**2**	BLASSO	Bayesian Lasso	*monomvn*
**3**	BRNN	Bayesian regularized neural networks	*brnn*
**4**	GBM	Stochastic gradient boosting	*gbm* & *plyr*
**5**	GLM	Generalized linear model	
**6**	GLMNET	Lasso and elastic-net regularized generalized linear models	*glmnet* & *Matrix*
**7**	GP-Poly	Gaussian process with polynomial kernel	*kernlab*
**8**	GP-Radial	Gaussian process with radial kernel	*kernlab*
**9**	KNN	k-nearest neighbors	*kknn*
**10**	LASSO	Lasso model	*elasticnet*
**11**	MARS	Multivariate adaptive regression spline	*earth*
**12**	MLR	Multivariate linear regression	
**13**	RF	Random forest	*randomForest*
**14**	RIDGE	Ridge regression	*elasticnet*
**15**	SVM-Radial	Support vector machines with linear kernel	*kernlab*
**16**	SVM-Linear	Support vector machines with radial kernel	*e1071*
Classification Models			

**1**	CART	Classification and regression trees	*rpart*
**2**	GBM	Stochastic gradient boosting	*gbm* & *plyr*
**3**	GLMNET	Lasso and elastic-net regularized generalized linear models	*glmnet* & *Matrix*
**4**	KNN	k-nearest neighbors	*kknn*
**5**	LDA	Linear discriminant analysis	*MASS*
**6**	LLDA	Localized linear discriminant analysis	*klaR*
**7**	MARS	Multivariate adaptive regression spline	*earth*
**8**	MDA	Mixture discriminant analysis	*mda*
**9**	NBC	Naive Bayes	*naivebayes*
**10**	NNET	Neural network	*nnet*
**11**	PDA	Penalized discriminant analysis	*mda*
**12**	PLS	Partial least squares	*pls*
**13**	RDA	Regularized discriminant analysis	*klaR*
**14**	RF	Random forest	*randomForest*
**15**	SVM-Linear	Support vector machines with linear kernel	*e1071*
**16**	SVM-Radial	Support vector machines with radial kernel	*kernlab*

Note: some models (e.g., support vector machines and random forest) can be used for both regression and classification purpose.

Supplementary Table 3. Example data sets used to test the functionality of HTPmod.

ID	Study / Reference	Data for modeling or visualization	Tested in	Supplementary Figures*
**1**	(Chen et al., 2014)[2]	HTP: using time-series image data to investigate plant growth and phenotypic components of drought responses in barley (*Hordeum vulgare*).	*growMod, htpdVis*	**Supplementary Figs. 3**, **5** and **11**
**2**	(Chen et al., 2018)[3]	HTP: using image-derived parameters to prediction plant biomass accumulation in three consecutive barley experiments.	*predMod, htpdVis*	**Supplementary Figs. 6-8** and **10**
**3**	(Jiao and Meyerowitz, 2010)[4] and (Chen et al.)	HTS: using ChIP-seq data to predict plant organ-specific gene expression patterns.	*predMod*	**Supplementary Fig. 9**;
**4**	(Fahlgren et al., 2015)[5]	HTP: using image data to predict plant growth under different water conditions in *Setaria*.	*growMod*	**Supplementary Figs. 4** and **5**
**5**	(Jiao and Meyerowitz, 2010)[4]	HTS: using TRAP-seq data to study flower organ-specific gene expression patterns in Arabidopsis.	*htpdVis*	**Fig. 4**
**6**	(Smaczniak et al., 2017)[6]	HTS: using SELEX-seq data to reflect the difference of DNA binding specificity of floral homeotic protein complexes and to further predict organ-specific target genes.	*htpdVis*	**Supplementary Fig. 12**
**7**	(Song et al., 2016)[7]	HTS: combining RNA-seq and ChIP-seq data to illuminate the relationship of differential gene expression patterns and the combinatorial regulation by multiple ABA-related TFs.	*predMod*	**Figs. 2 and 3; Supplementary Fig. 7**

**8**	(Wang et al., 2015)[8]	HTS: use Hi-C and ChIP-seq data to describe chromatin states in Arabidopsis	*htpdVis*	**Supplementary Fig. 13**
**9**	(Zhu et al., 2018)[9]	HTP: profiling of 980 metabolites in 442 tomato (*Solanum lycopersicum*) accessions.	*htpdvis*	**Supplementary Fig. 13**

[*] Note: reanalysis of published data by HTPmod (see online document for how data were collected). Results are shown in the Supplementary Figures as below. Although the examples were selected from studies in plants, HTPmod can broadly be used for studies in any other organisms. HTP: high-throughput phenotyping; HTS: high-throughput sequencing.

Supplementary Figures

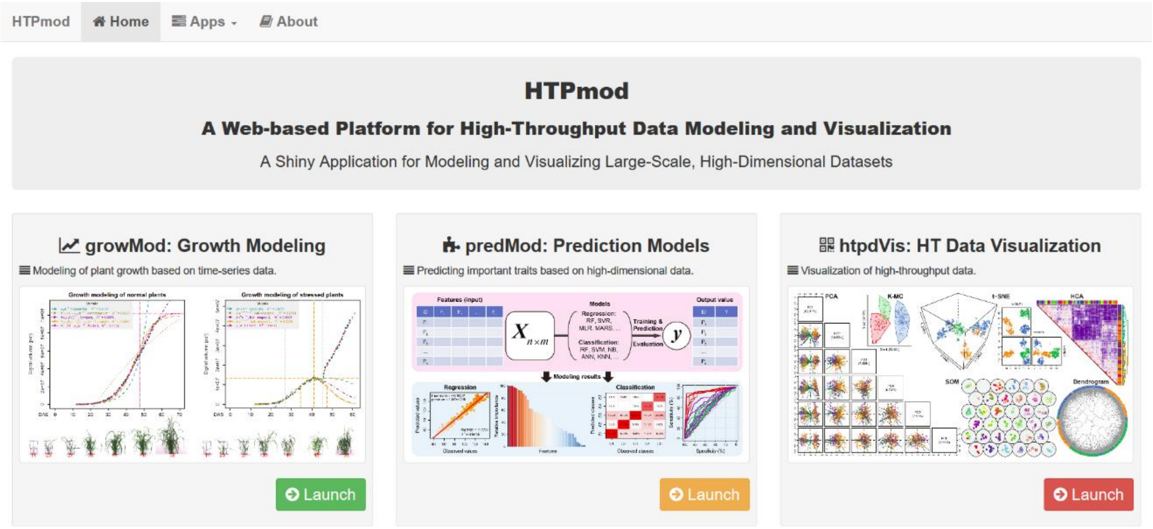

Supplementary Figure 1. Screenshot of the homepage of HTPmod.

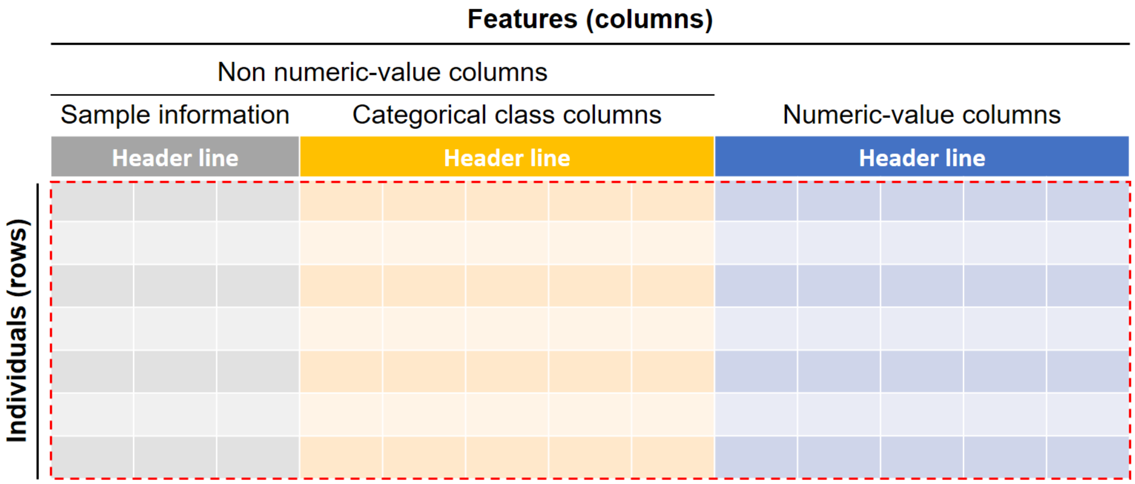

Supplementary Figure 2. HTPmod accepts the simplest tables files (with rows as individuals and columns as features; the header line is required) as the only input. In the modules of *predMod* and *htpdVis*, HTPmod automatically determine the column types according their contents: columns containing non numeric values are considered as either annotation for individuals (in grey) or categorical features (in orange). Categorical class columns can be either used for color schema purpose in plots or the output feature in the classification analysis. Users need to convert

numeric categorical features into non-numeric values if input files containing numeric values for categorical purposes.

In the subsections, we demonstrated how to use HTPmod functionalities to explore high-throughput datasets from the selected studies in **Supplementary Table 3**.

1. Growth modeling with the *growMod* module

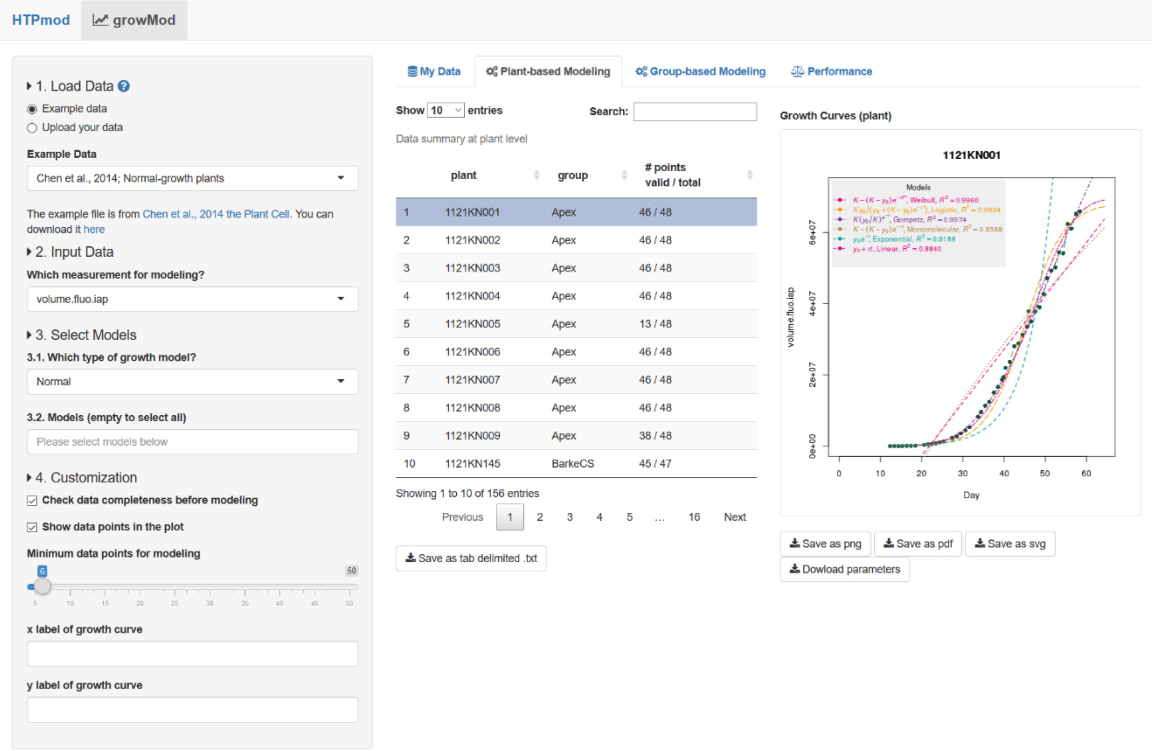

Supplementary Figure 3. Screenshot of the *growMod* module. Example shows the growth modeling of barley plants under normal growth conditions[2].

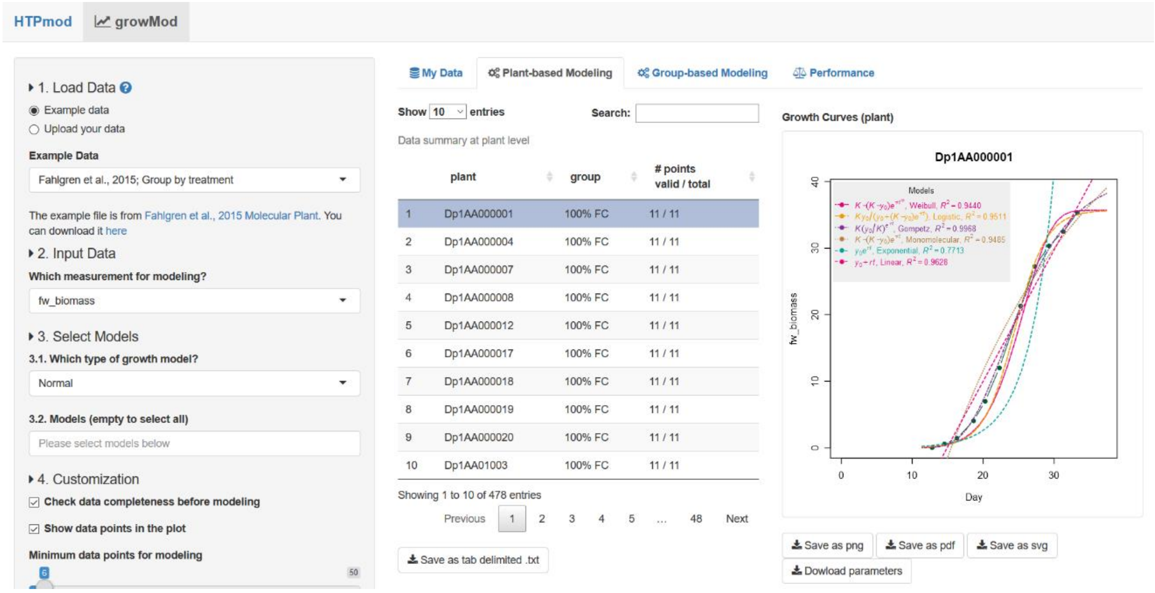

Supplementary Figure 4. Growth modeling of *Setaria* plants[2].

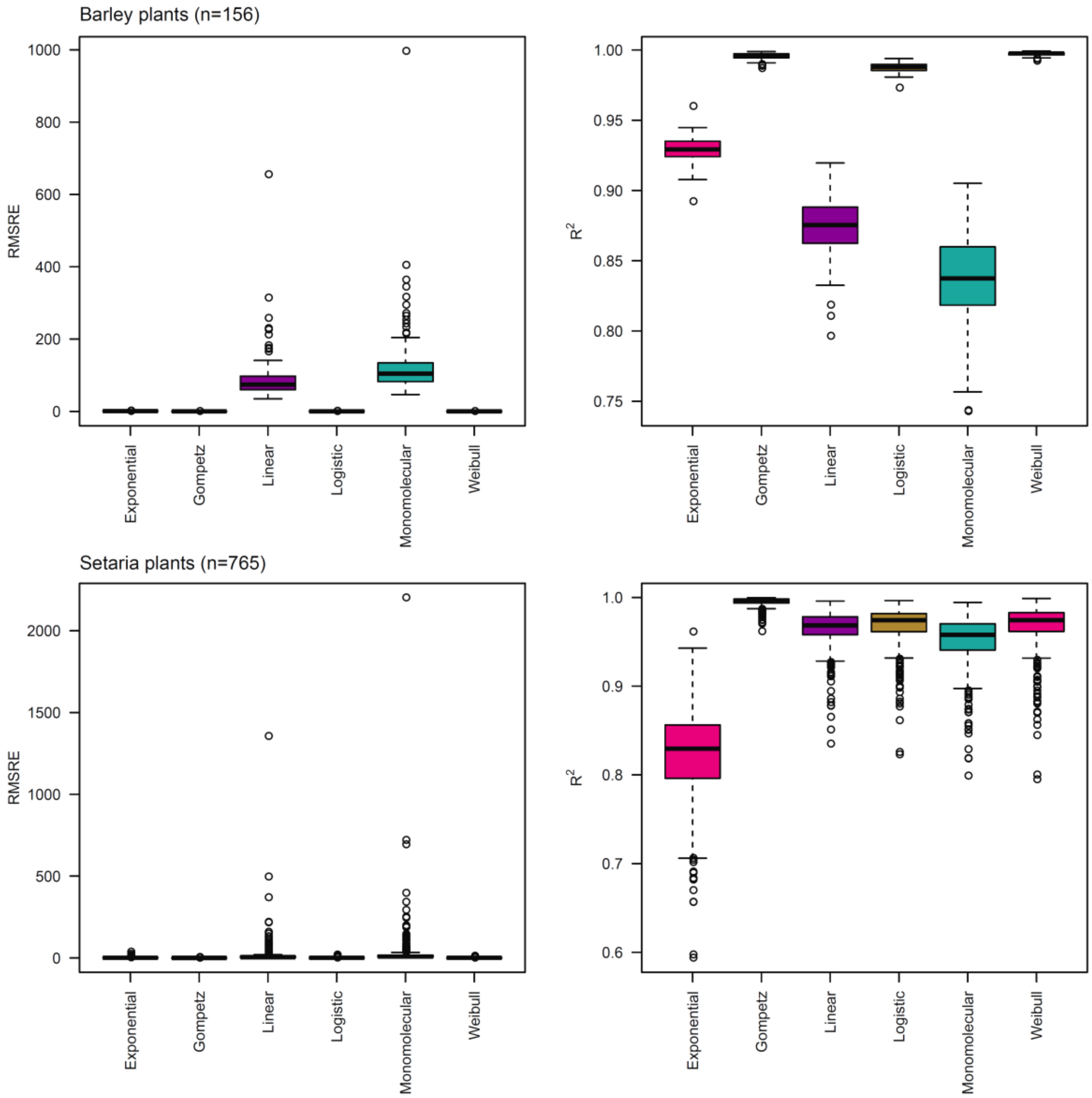

Supplementary Figure 5. Performance of growth models in barley[2] (top panel; using the example data “Chen et al., 2014; Normal-growth plants”) and Setaria[5] (bottom panel; using the example data “Fahlgren et al., 2015; Group by genotype”). Results showed that Weibull model shows the best performance for modeling growth of barley plants while Gompetz model does for Setaria plants. Default parameter settings under the “Performance” page were used in the two analyses.

2. Prediction models: the *predMod* module

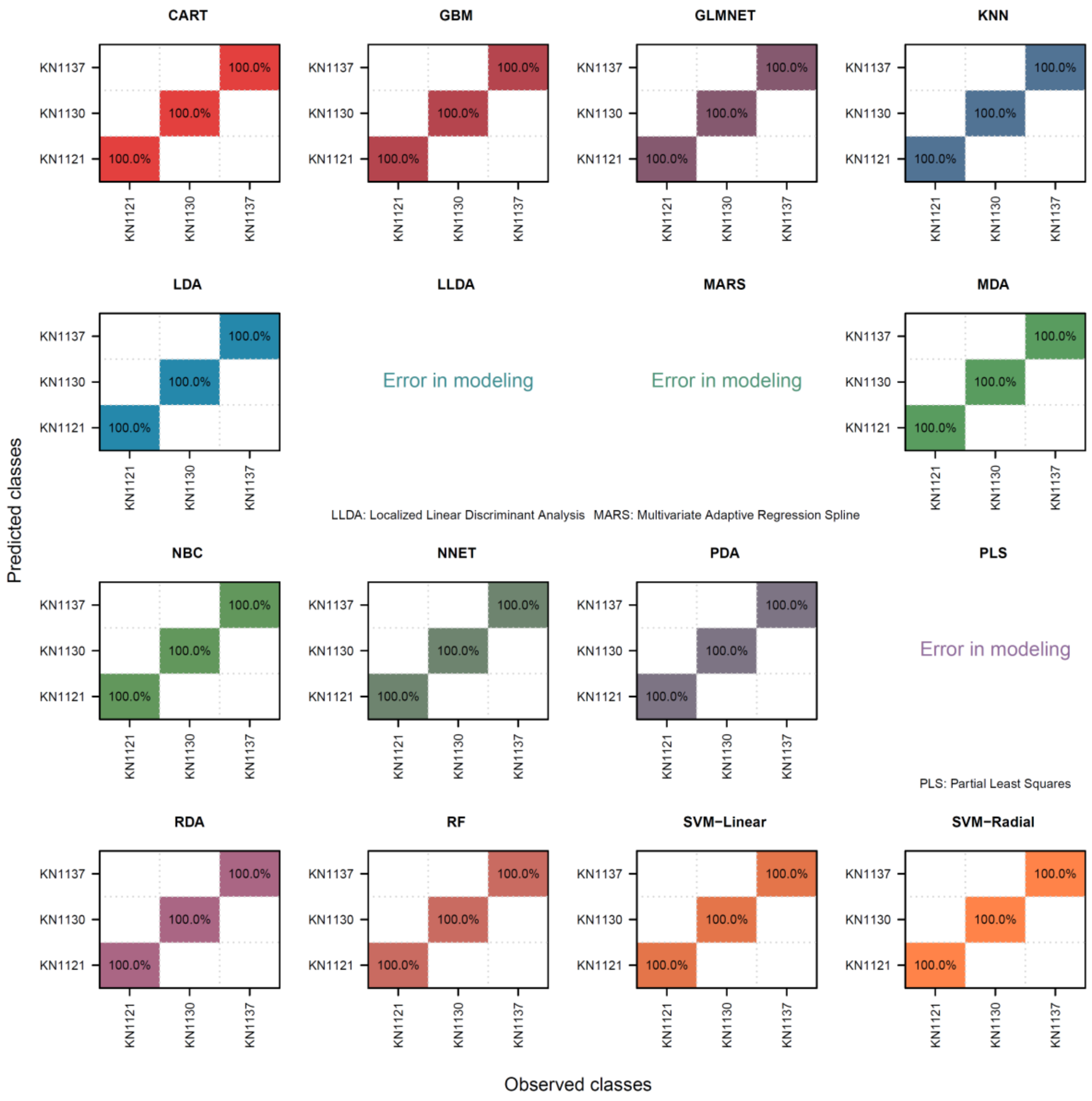

Supplementary Figure 6A. Classification of three consecutive HTP experiments[3] based on image-derived features (the example data "Chen et al., 2018; Regression or Classification"). Note that some models were failed to run on this dataset; all other models perfectly separated the experiments. Default parameter settings for all the classification models were used in the analysis.

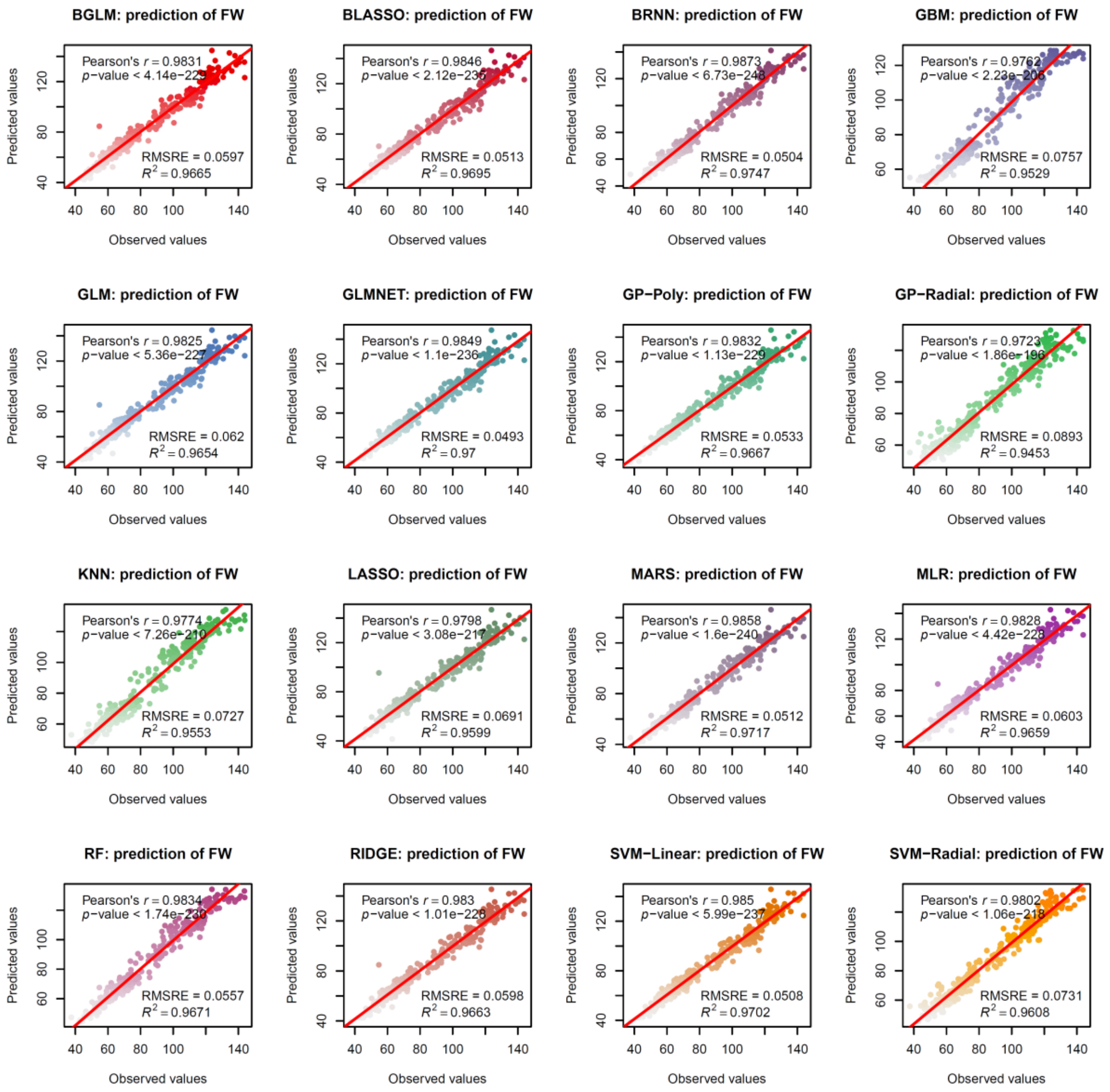

Supplementary Figure 6B. Prediction of plant biomass accumulation with image-derived parameters, using HTP data from ref[3] (the example data "Chen et al., 2018; Regression or Classification"). Each panel shows the prediction result for a regression model. See **Supplementary Table 2** for the full model names. Default parameter settings for all the regression models were used in the analysis.

Supplementary Figure 6. (**A-B**) Apply various prediction models on HTP data.

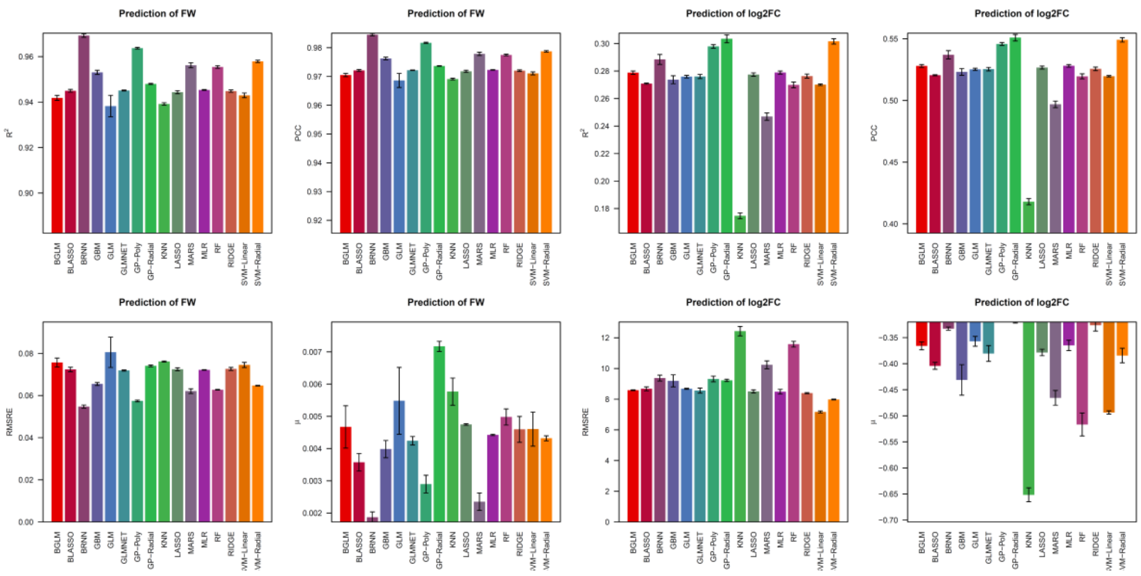

Supplementary Figure 7. Evaluation of the model performance in the prediction of plant biomass based on image-derived paramters[3] (left; the example data "Chen et al., 2018; Regression or Classification") or gene expression changes based on transcription factor binding data[7] (right; the example data "Song et al., 2016; Regression"). All parameters were set in default in the analysis.

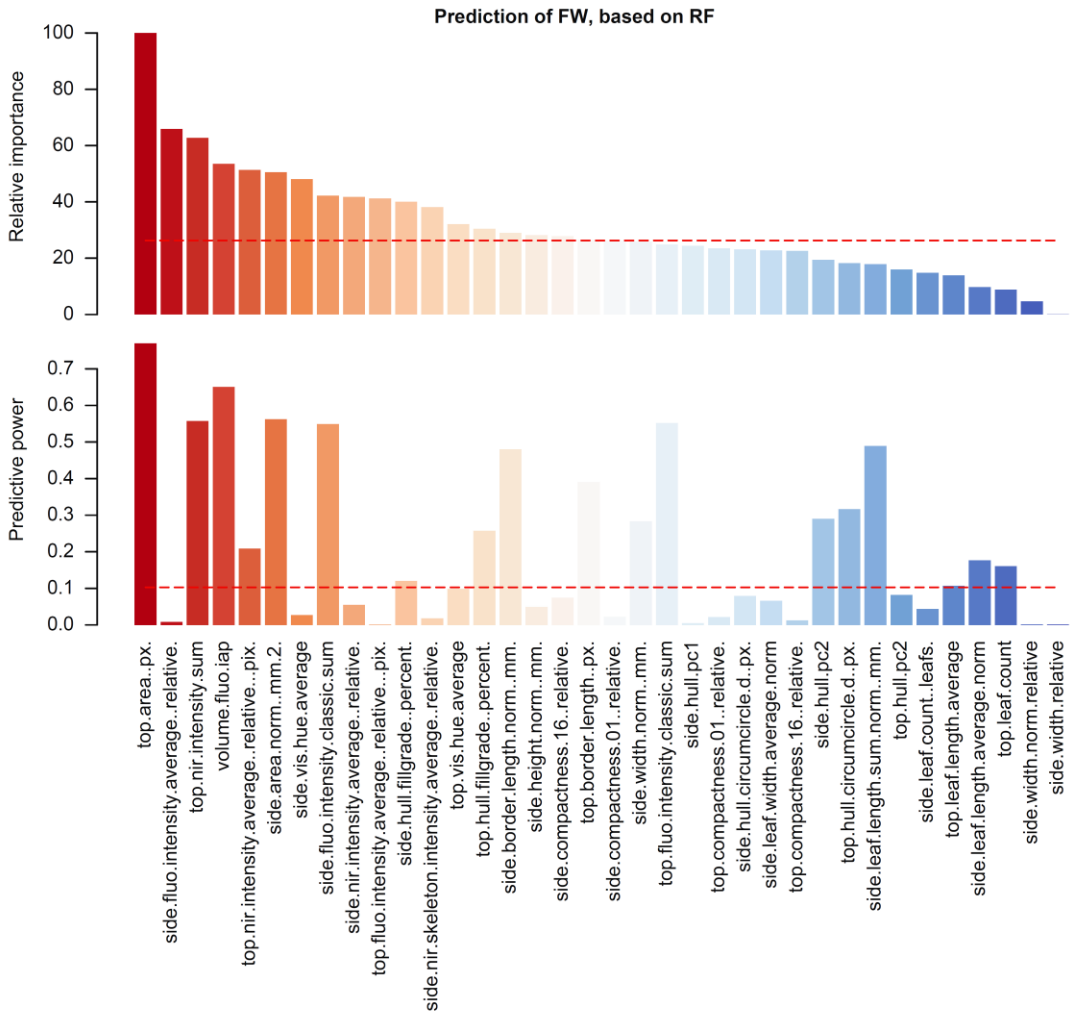

Supplementary Figure 8. Calculate the relative importance of features in regression models. Random forest (RF) model was used for prediction of barley biomass accumulation, using image-derived feature data in three consecutive HTP experiments[3] (the example data "Chen et al., 2018; Regression or Classification"). The results are consistent to that in the original study.

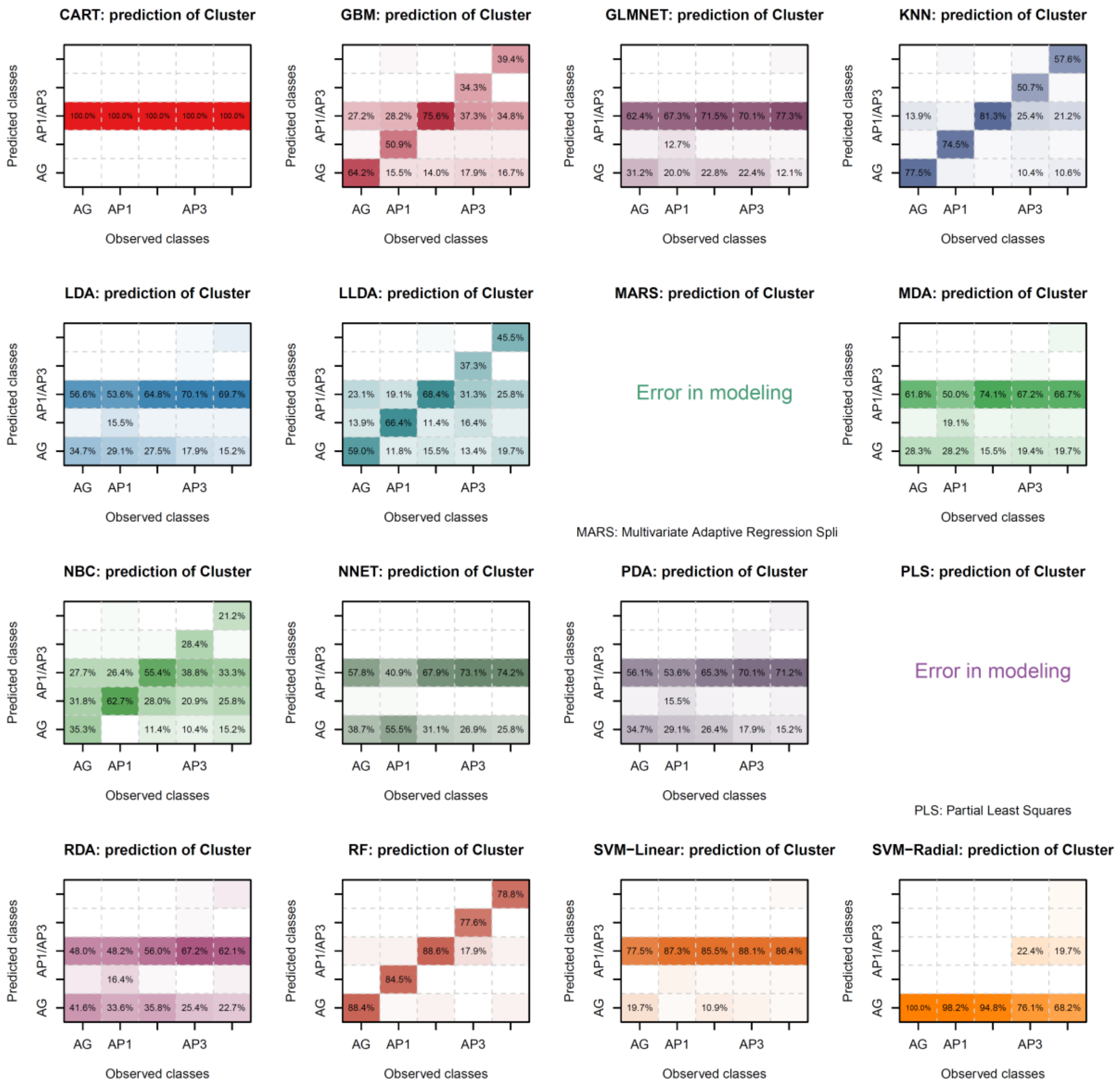

Supplementary Figure 9A. Confusion matrixes for measuring the classification accuracy of different models. Note that some models were failed to run on this dataset.

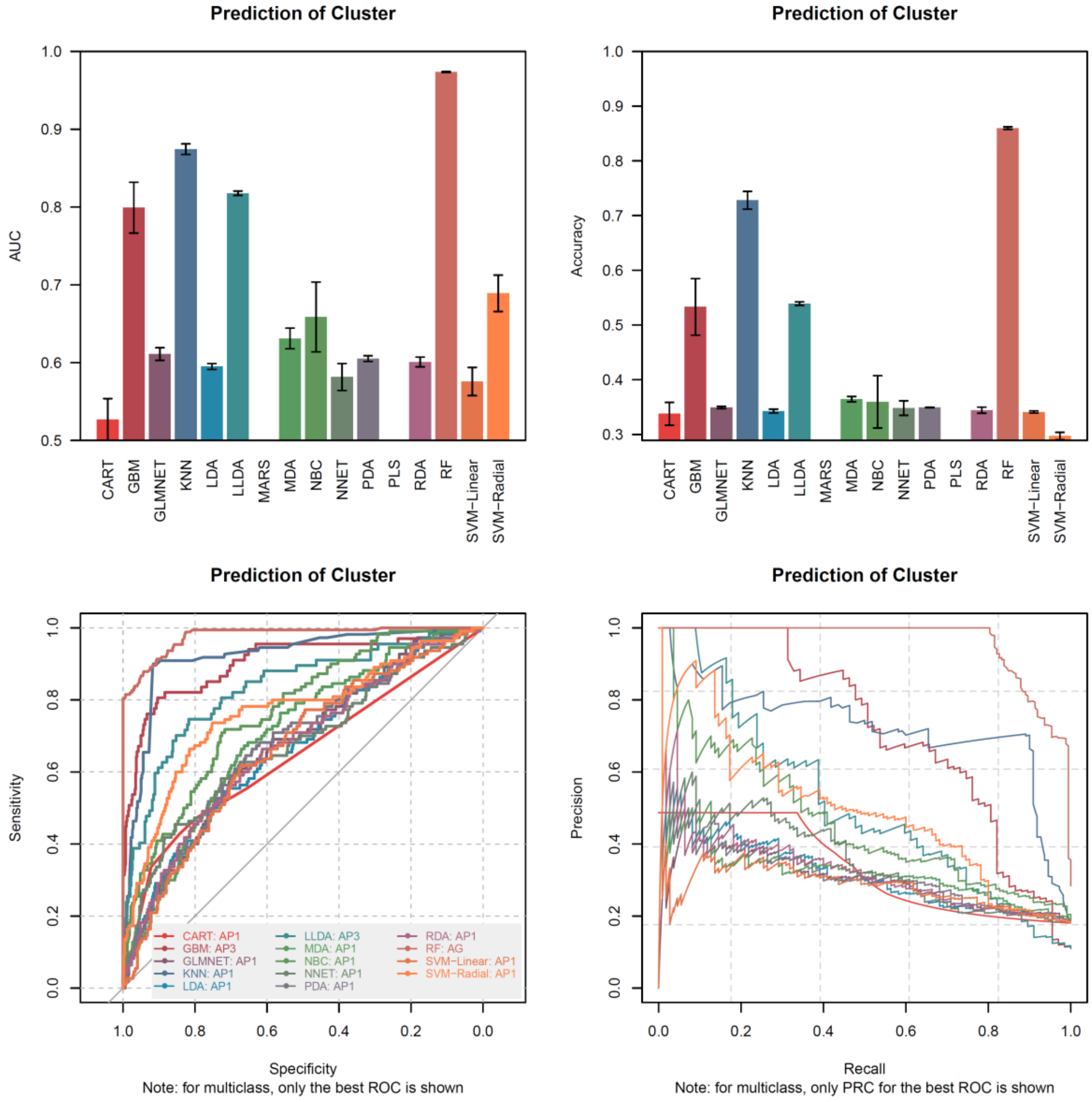

Supplementary Figure 9B. Evaluation of the performance of different classification models. Results show that RF (random forest) and KNN (k-nearest neighbors) outperform other models.

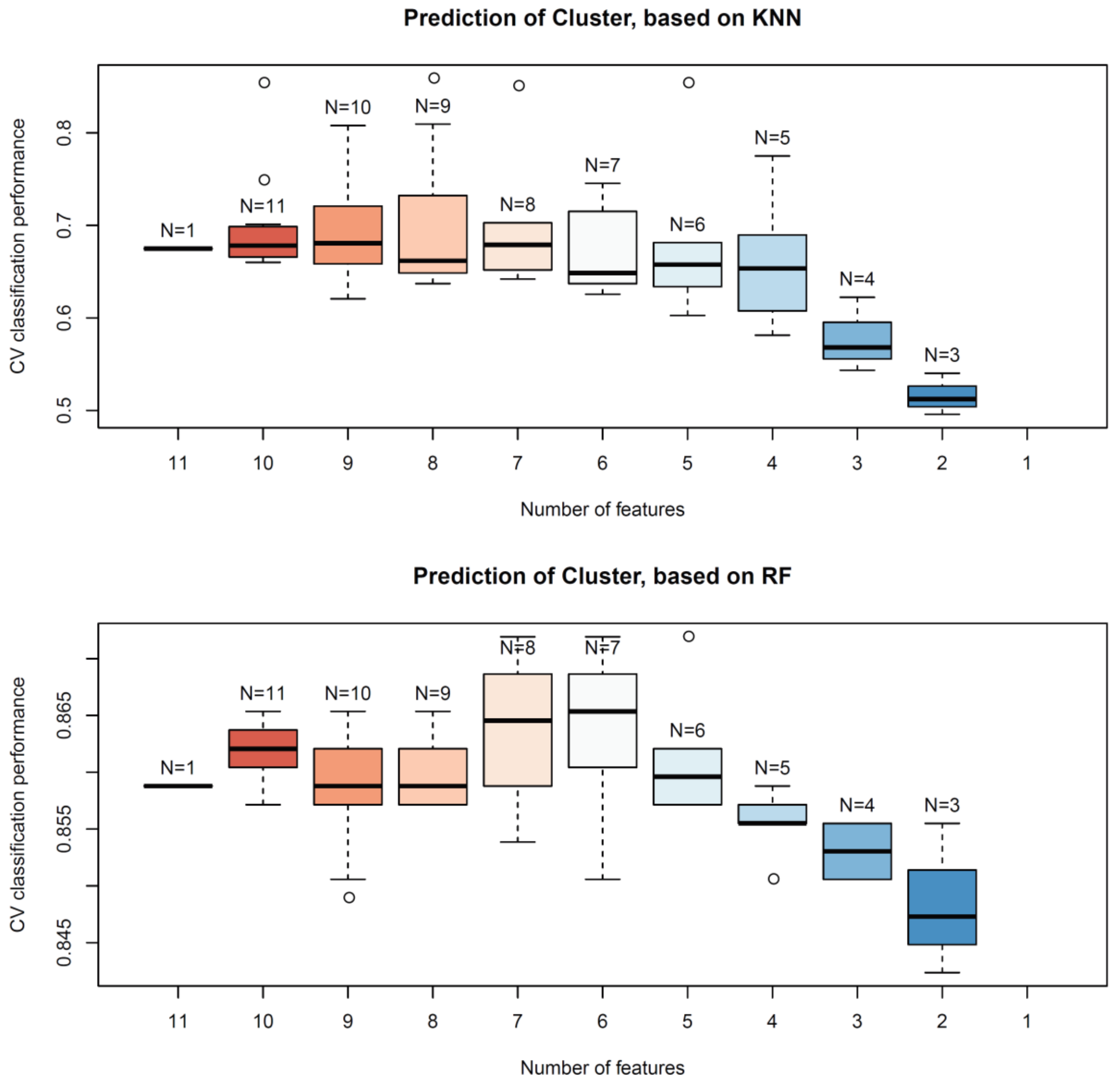

Supplementary Figure 9C. Influence of feature selection on classification performance. Results from both models show that at least four features are required to retain good classification performance. This suggests that (1) plant organ differentiation is not controlled by a single factor, but rather depends on the joint behavior of multiple factors and (2) that there might be redundancy between factors.

Supplementary Figure 9. (**A-C**) Apply classification models to predict plant organ-specific gene expression patterns (using the example data "Chen et al., 2018; Classification"). All parameters were set as default in the analysis.

3. High-throughput data visualization with the *htpdVis* module

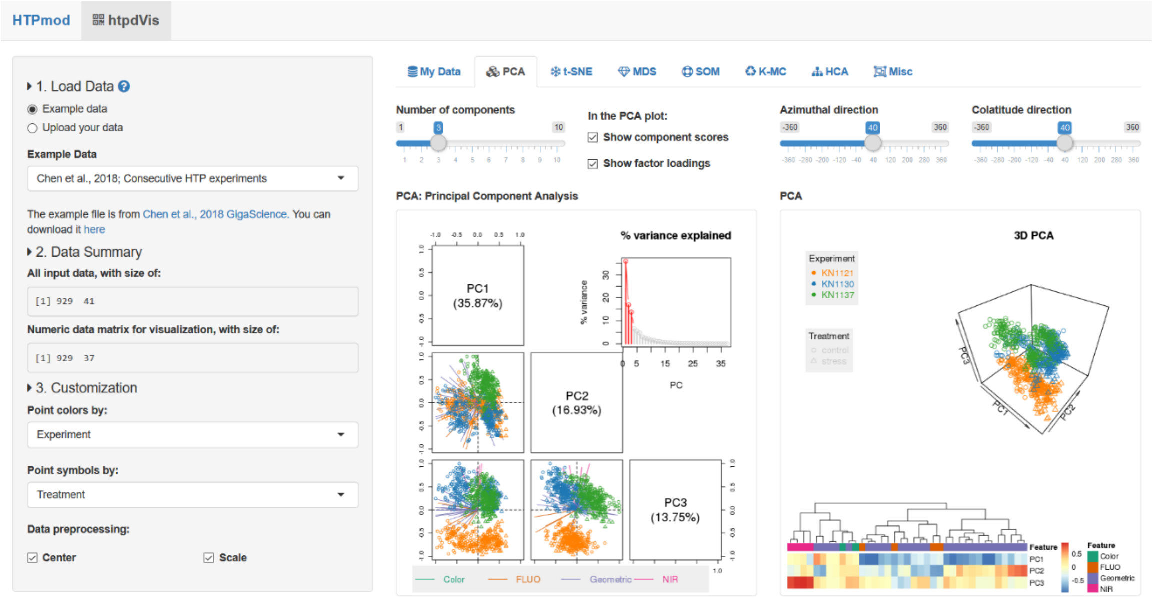

Supplementary Figure 10A. Principal component analysis (PCA).

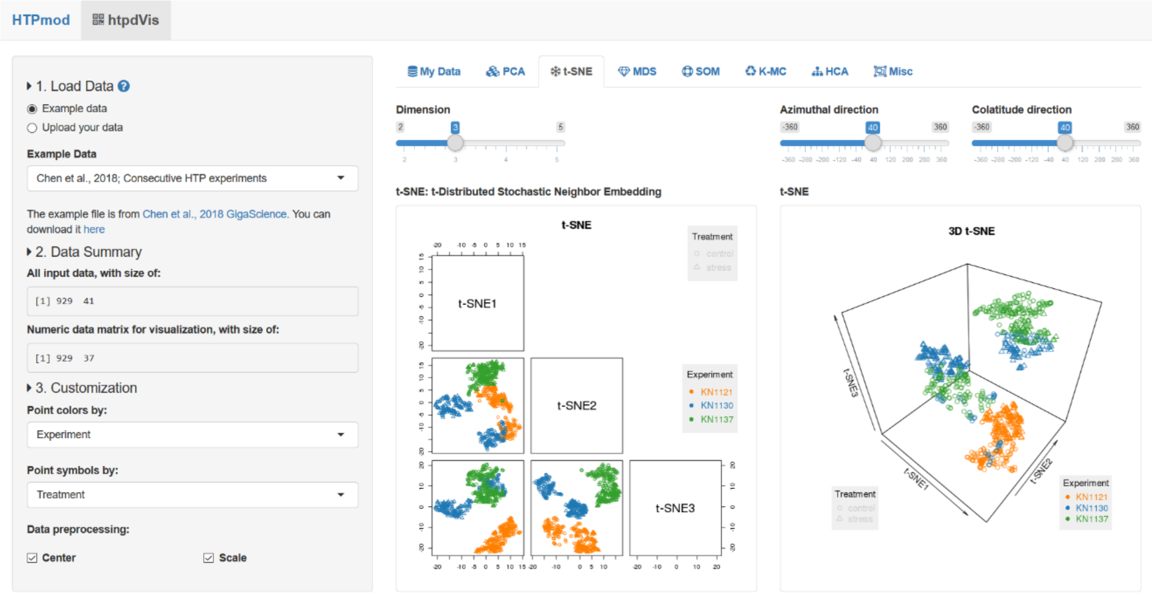

Supplementary Figure 10B. t-distributed stochastic neighbor embedding (t-SNE).

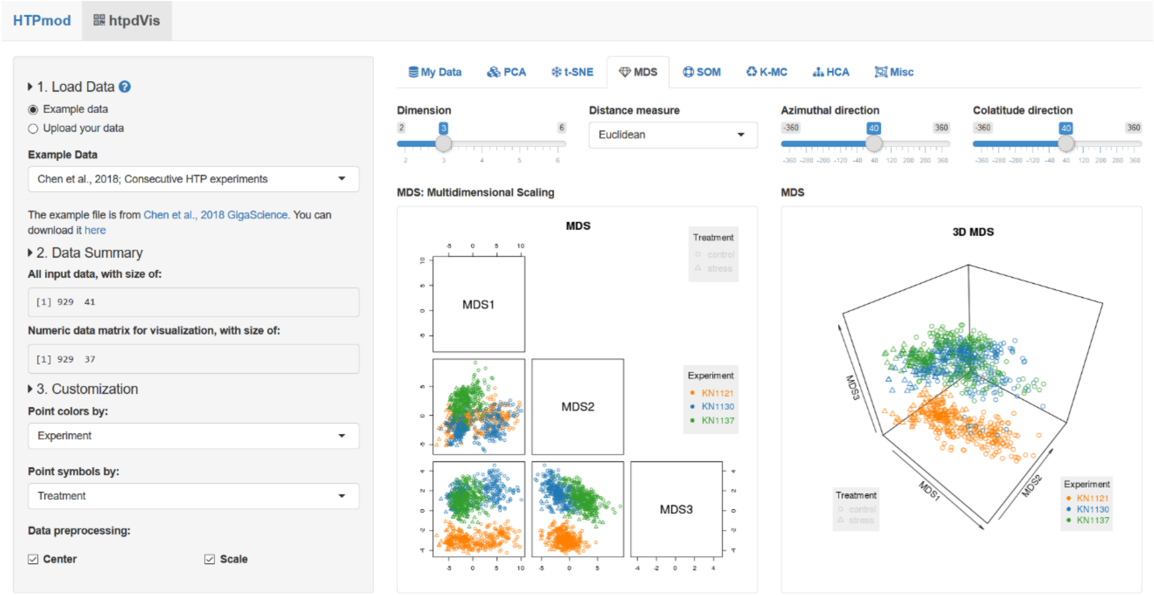

Supplementary Figure 10C. Multidimensional scaling (MDS).

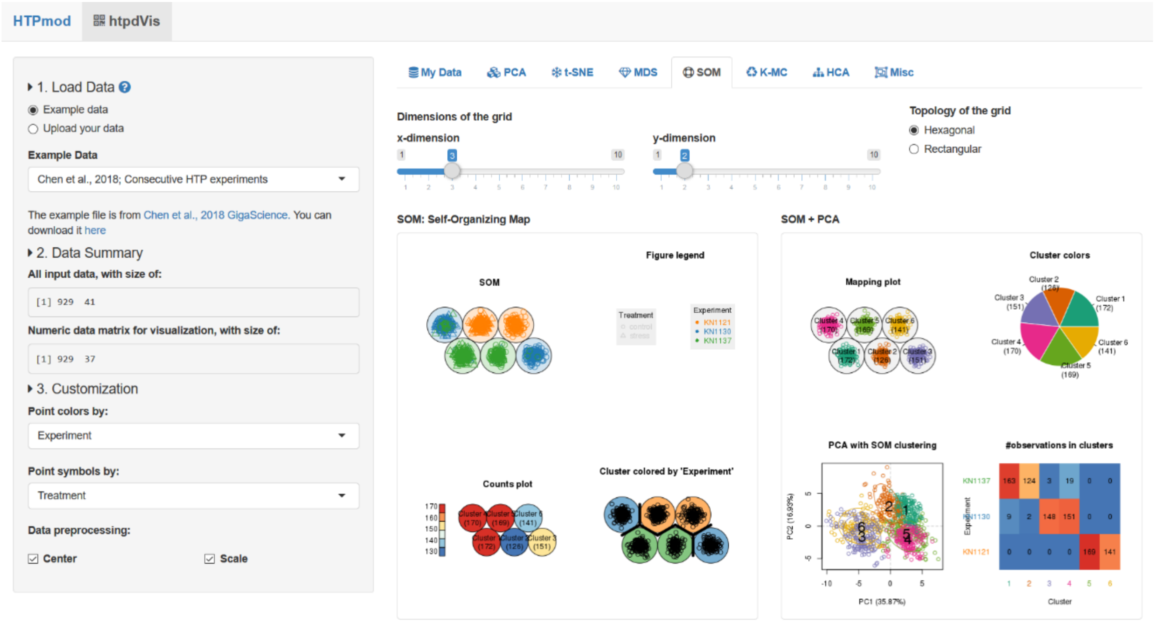

Supplementary Figure 10D. Self-organizing map (SOM).

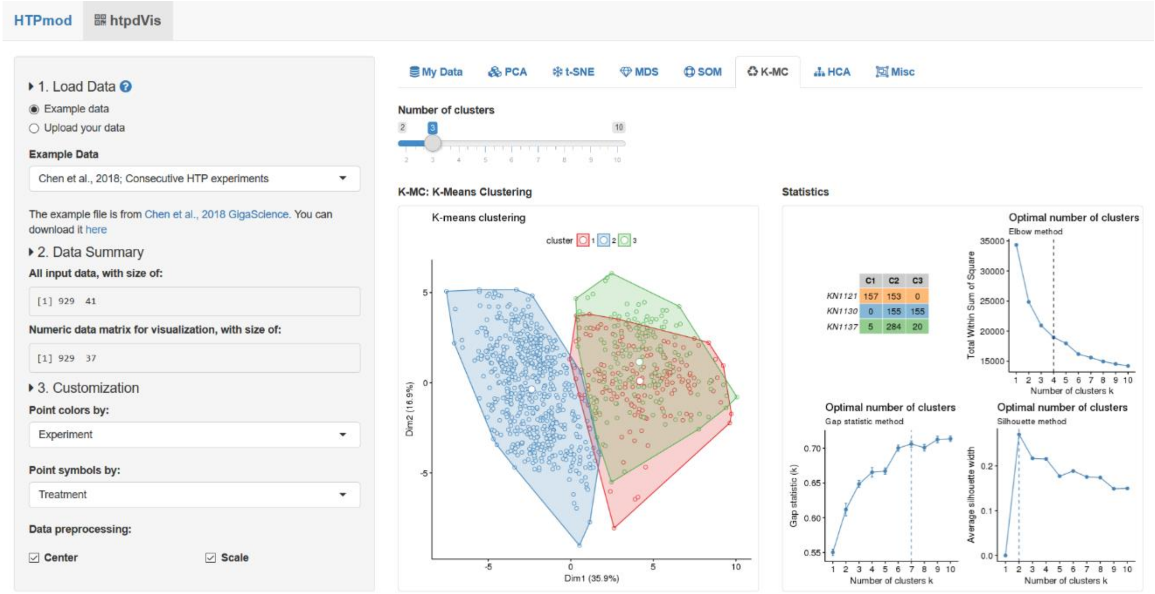

Supplementary Figure 10E. K-means clustering (K-MC).

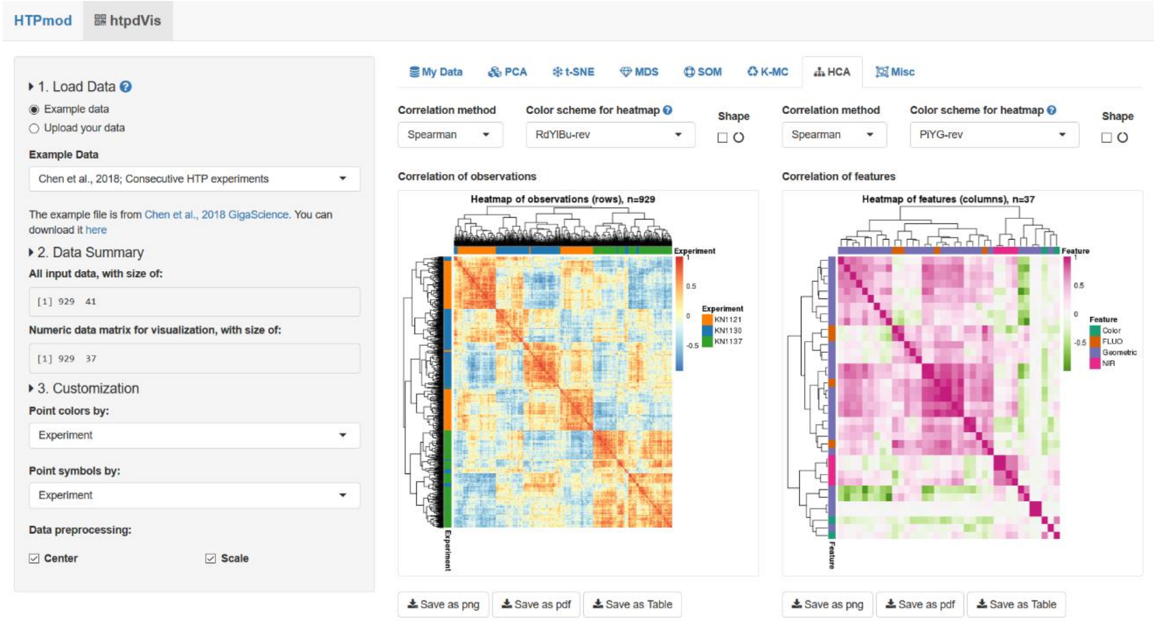

Supplementary Figure 10F. Hierarchical cluster analysis (HCA) with heatmaps.

Supplementary Figure 10. (**A-F**) Apply different visualization tools to the same dataset obtained from ref[3] (i.e., the example data "Chen et al., 2018; Consecutive HTP experiments").

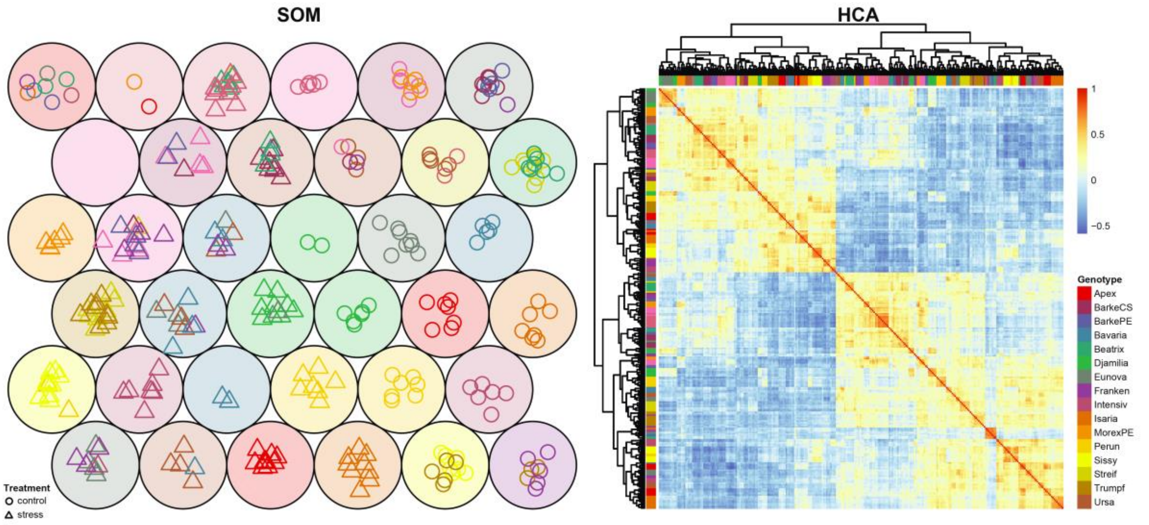

Supplementary Figure 11. Reanalysis of the HTP data from ref[2] (i.e., the example data "Chen et al., 2014; Barley HTP data"). Plants in the same genotypes under the same growth condition show similar phenotypic profile, as show in SOM (left; 6x6; colored by "Genotype" and shaped by "Treatment") and HCA (right; bars colored by "Genotype").

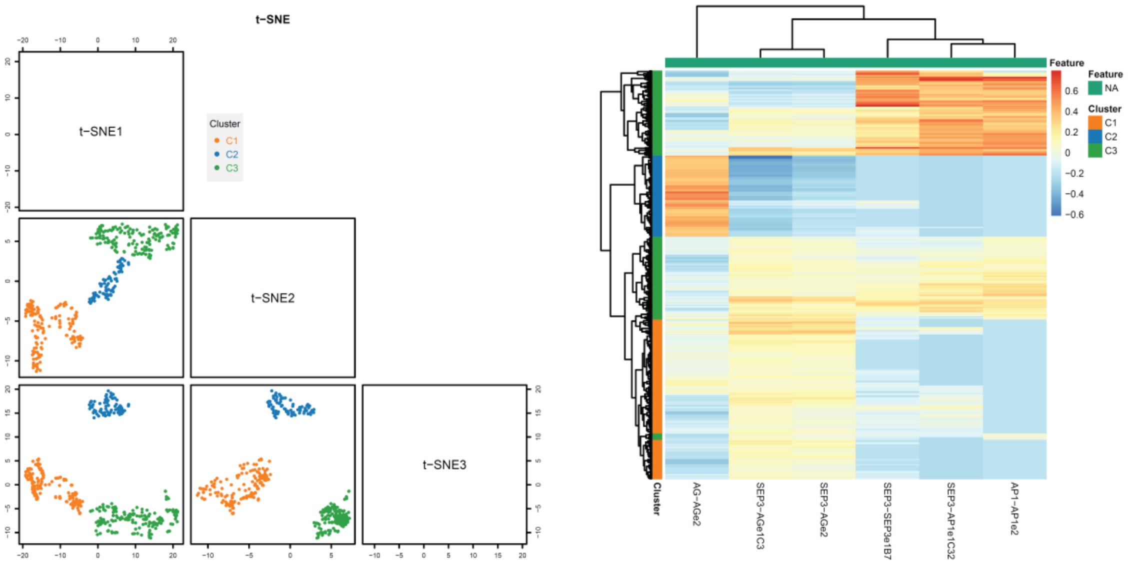

Supplementary Figure 12. Visualize the DNA binding specificity (based on SELEX-seq data) of floral homeotic protein complexes[6] (the example data "Smaczniak et al., 2017; DNA binding specificity of MADS proteins") using t-SNE (left; dimension=3, data points colored by "Cluster") and HCA (right; left bars colored by "Cluster").

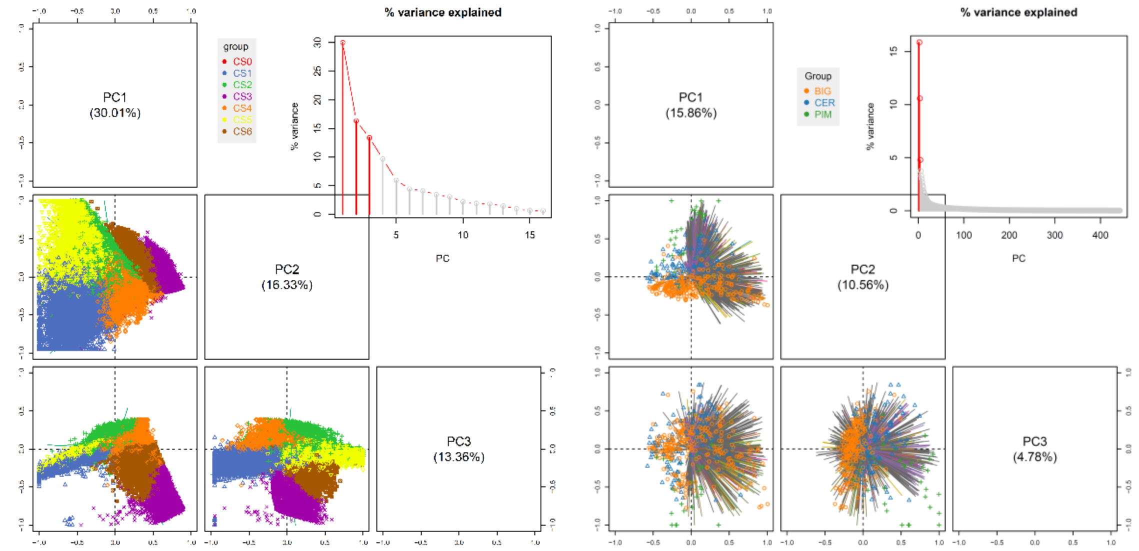

Supplementary Figure 13. PCA on chromatin states identified by 16 epigenetic data sets over the Arabidopsis epigenome at 400-bp resolution[8] (left; the example data "Wang et al., 2015; Chromatin states") or a metabolome dataset with profiling data of 980 metabolites in 442 tomato accessions[9] (right; the example data "Zhu et al., 2018; Tomato metabolomes"). Results are similar to the original studies.

Supplementary References

1. Chen, D. Dissecting and Modeling the Phenotypic Components of Plant Growth and Drought Responses Based on High-throughput Image Analysis. *(Doctoral Diss. Martin-Luther-Universität Halle-Wittenberg)* (2017).

2. Chen, D. *et al.* Dissecting the phenotypic components of crop plant growth and drought responses based on high-throughput image analysis. *Plant Cell* **26,** 4636–4655 (2014).

3. Chen, D. *et al.* Predicting plant biomass accumulation from image-derived parameters. *Gigascience* (2018). doi:10.1093/gigascience/giy001

4. Jiao, Y. & Meyerowitz, E. M. Cell-type specific analysis of translating RNAs in developing flowers reveals new levels of control. *Mol. Syst. Biol.* **6,** (2010).

5. Fahlgren, N. *et al.* A versatile phenotyping system and analytics platform reveals diverse temporal responses to water availability in Setaria. *Mol. Plant* **8,** 1520–1535 (2015).

6. Smaczniak, C., Muiño, J. M., Chen, D., Angenent, G. C. & Kaufmann, K. Differences in DNA-binding specificity of floral homeotic protein complexes predict organ-specific target genes. *Plant Cell* **29,** tpc.00145.2017 (2017).

7. Song, L. *et al.* A transcription factor hierarchy defines an environmental stress response network. *Science (80-.).* **354,** aag1550-aag1550 (2016).

8. Wang, C. *et al.* Genome-wide analysis of local chromatin packing in Arabidopsis thaliana. *Genome Res.* **25,** 246–256 (2015).

9. Zhu, G. *et al.* Rewiring of the Fruit Metabolome in Tomato Breeding. *Cell* **172,** 249–261.e12 (2018).
